# Strategies, debates, and adversarial collaboration in working memory: The 51st Bartlett Lecture

**DOI:** 10.1177/17470218231194037

**Published:** 2023-08-23

**Authors:** Robert H Logie

**Affiliations:** University of Edinburgh, Edinburgh, UK

**Keywords:** Working memory, adversarial collaboration, cognitive strategies, resolving scientific debates

## Abstract

Frederic Bartlett championed the importance of individual strategy differences when remembering details of events. I will describe how long-running theoretical debates in the area of working memory may be resolved by considering differences across participants in the strategies that they use when performing cognitive tasks, and through adversarial collaboration between rival laboratories. In common with the established view within experimental cognitive psychology, I assume that adults have a range of cognitive functions, evolved for everyday life. However, I will present evidence showing that these functions can be engaged selectively for laboratory tasks, and that how they are deployed may differ between and within individuals for the same task. Reliance on aggregate data, while treating inter- and intra-participant variability in data patterns as statistical noise, may lead to misleading conclusions about theoretical principles of cognition, and of working memory in particular. Moreover, different theoretical perspectives may be focused on different levels of explanation and different theoretical goals rather than being mutually incompatible. Yet researchers from contrasting theoretical frameworks pursue science as a competition, rarely do researchers from competing labs work in collaboration, and debates self-perpetuate. These approaches to research can stall debate resolution and generate ever-increasing scientific diversity rather than scientific progress. The article concludes by describing a recent extended adversarial collaboration (the WoMAAC project) focused on theoretical contrasts in working memory, and illustrates how this approach to conducting research may help resolve scientific debate and facilitate scientific advance.

## Understanding human memory

Something happens in individuals following the offset of an observed event that allows them subsequently to carry out event-related activities such as making a decision, reporting details, performing an action, or noting similarities with an earlier or later event. The time gap between the initial event and the individual action can be from milliseconds to decades, providing evidence of an ability to retain and to retrieve features of past events. In the psychology laboratory and in the world at large this ability is referred to as “memory,” suggesting that by giving it a label we know what it is. In the experimental psychology laboratory, the widespread assumption is that there are general principles as to the functional organisation of memory, even if its contents vary from one individual to another, vary across the lifetime of each individual, and vary over different timescales for retention. All we need to do is run the right experiments and those general principles will emerge. There are some general principles that have emerged, notably that not all details of an event are retained, many details that are available after short intervals are no longer available after longer intervals or after additional events, and the subset of details that are retained from multiple events accumulate to allow for learning and understanding the world around us. However, this much has been known for some time. For example, John Locke (1690) viewed one aspect of “retention” (memory) as:

. . . the power to revive again in our minds those ideas, which after imprinting, have disappeared, or have been, as it were, laid out of sight . . . the storehouse of our ideas . . . There is an ability of the mind . . . to revive them again . . . though some with more, some with less difficulty; some more lively, and others more obscurely.

This refers to what is broadly termed “long-term memory,” to include stored knowledge (semantic memory), practised actions (procedural memory), and representations of specific events (episodic memory). Locke also referred to the contrasting concept of *contemplation* as “. . . keeping the idea, which is brought into it, for some time actually in view.” We might interpret the latter as akin to one key aspect of what is now called working memory.

A large volume of experimental studies, not least by Bartlett (1932/1961), has led to broad agreement among researchers on the Locke (1690) characterisations of memory and of what he called contemplation. However, over two centuries later there are long-running major debates regarding the details. We do understand much better, at both the cognitive and neurobiological levels, what determines the processing of Locke’s *imprinting* (now usually called encoding), many characteristics of the *storehouse*, and many of the factors that influence whether memories are *revived with more or less difficulty* (see reviews in Baddeley et al., 2020). Here, I will focus more on contemporary debates regarding Locke’s suggestion about *contemplation*, referred to by William James (1902, p. 645) as *primary memory* and the *specious present* (for a historical review see Logie, 1996). For both of those early authors the concepts of contemplation and the specious present were derived from what James (1902, p. 644) called *introspective psychologizing* regarding their own cognition, rather than objective observations of others in experiments. Over a century later, accumulation of a large volume of experimental evidence on working memory has led most contemporary researchers on this topic to view working memory as broadly consistent with *contemplation* and the *specious present* from the early *introspective psychologizing*, by referring to the ability to keep a small amount of information readily available to support current activities such as reasoning and decision making, guiding actions, keeping track of conversations, navigating, and updating our mental representation of rapid changes in the environment (Logie et al., 2021a). However, there are multiple ongoing, and long-lasting debates regarding the details of how the concept of working memory functions to support these activities (for contrasting reviews see Logie et al., 2021b).

In this article, first, there will be a brief overview of the development of theoretical perspectives regarding working memory, and of the rapid growth in the number of different perspectives and ongoing unresolved debates. This will lead to the proposal that contrasting assumptions and debates in this field might have arisen, at least in part, from reliance on data aggregated across participants in experiments. Evidence for individual variation in the use of cognitive strategies for the same tasks challenges the assumption that general principles of memory can be derived from patterns of aggregate data. Following Bartlett (1932), the discussion will argue that a focus on individual differences in how each participant performs laboratory tasks (not only individual differences in performance levels) offers a more fruitful approach to exploring general principles of memory. Examples will be drawn from studies of healthy adults and case studies of memory impairments following focal brain damage. This will lead on to suggesting that some of the debates between working memory theories may be more apparent than real, that competitive theorising rarely leads to debate resolution, and that the proliferation of theories and self-perpetuation of debates act to inhibit scientific progress. The approach of adversarial collaboration (e.g., Clark et al., 2022; Cowan et al., 2020; Kahneman, 2003; Logie, Belletier, & Doherty, 2021) will be proposed as a means to resolve debates that otherwise self-perpetuate without generally agreed advances in understanding. The discussion will conclude by arguing for debates that actively seek greater theoretical and empirical integration, rather than ever-increasing and unresolved diversity, to accelerate scientific advance.

## Working memory: development of debates

The idea that working memory is not only a temporary, limited capacity memory store, but involves a range of operations that can manipulate the contents of that store, was addressed by Atkinson and Shiffrin (1968), who presented empirical evidence and a theoretical argument for precisely this idea. They proposed a multiple component system for human memory, comprising a short-term store for auditory–verbal–linguistic (a-v-l store) material and a range of control processes, in addition to a sensory visual store of very brief duration (<1,000 ms), a range of additional sensory stores, and a long-term store. Most of the research reviewed by Atkinson and Shiffrin focused on memory for verbal material, such as lists of words or of pairs of words, and they noted the evidence (e.g., Conrad, 1964) suggesting that visually presented words tend to be stored on the basis of how they sound when pronounced (phonology). They also noted studies of individuals with temporary or permanent brain damage, primarily to the hippocampus, who showed largely intact functioning of short-term or working memory, yet after delays of more than a few minutes were unable to retrieve details of experimental materials or of events, or of people that they did not know before the damage occurred (e.g., Barbizet, 1963; Milner, 1959, 1967; see also Corkin, 2013; Milner, 1971; Milner et al., 1968; recent reviews in MacPherson & Della Sala, 2019). These findings, and other evidence from studies of healthy adults, were used to support their view that the short-term store plus sensory stores, and control processes could be considered as separate from the long-term memory system that can be selectively impaired, even if the sensory, short-term, and long-term systems interact seamlessly in the healthy brain. Moreover, control processes could be selected by the participant, depending on their strategy for performing a given task. Finally, Atkinson and Shiffrin argued that information from visual input was held briefly in the visual sensory store: control processes translated this into an auditory–verbal code for the a-v-l store where it was maintained by rehearsal, while being copied into long-term memory. There were suggestions of the possibility of a range of other modality specific stores, notably a short-term visual memory, but this was not pursued in any detail. The authors also raised the possibility of direct transfer from sensory input into the long-term store, but again, this was not pursued. Their key proposal for the flow of information was that working memory (comprising one or more short-term stores and control processes) acted as a route between sensory input and long-term memory, while also receiving previously stored information from long-term memory. For recent comments on the influence of Atkinson and Shiffrin (1968), see Malmberg et al. (2019).

The idea of a multiple component model focused on working memory was developed further by Baddeley and Hitch (1974; Baddeley, 1986), who provided more detailed evidence for an auditory–verbal store that was viewed as a component of a limited capacity workspace, referred to as the articulatory loop, and not the core of working memory as suggested by Atkinson and Shiffrin (1968). Specifically, the articulatory loop was assumed to provide limited capacity temporary verbal storage, and was not the same system for control process that are involved in, for example, reasoning or language comprehension. This was tested by asking participants to store sequences of random letters or digits, next to perform a reasoning or comprehension task during a memory retention interval, and then to recall the letters or digits. This is known as dual-task methodology. If performance on either the memory task and/or the reasoning/comprehension task is poorer when performing both together (dual task) compared with performing each task on its own (single task), this would indicate that they share the same underlying system. However, if there is no difference in performance between single- and dual-task conditions, this would indicate that they rely on separate systems that can act in concert. Baddeley and Hitch found that with a memory load of three letters or digits, then neither recall of the verbal sequence, nor a reasoning task or comprehension task performed during the retention interval were affected compared with single-task conditions. With a sequence of six items to remember, dual task reduced the number of items that could be recalled, and reduced performance on the reasoning or comprehension task. These results pointed to the conclusion that a temporary verbal store has the capacity to retain at least three items, while some other part of working memory is engaged with reasoning or comprehension during the retention interval. When the capacity of the temporary memory system is exceeded (six items), another component of working memory is required to support memory for the additional items, but that component is also required for reasoning or comprehension. The articulatory loop (later the phonological loop, e.g., Baddeley et al., 1998) was assumed to rely primarily on phonological and articulatory coding and could retain three or four words, letters, or numbers for around 2 s. Retention of the verbal sequence could be extended for longer than 2 s by continued repetition with verbal rehearsal. Atkinson and Shiffrin viewed mental verbal rehearsal as a control process to transfer information from the short-term store into the long-term memory. In contrast, Baddeley and Hitch viewed verbal rehearsal, not as support for long-term learning, but as a means to maintain a small amount of verbal information on a temporary basis. This latter view was consistent with other work showing that repeated rehearsal does not necessarily lead to long-term learning (e.g., Rundus, 1971). Baddeley and Hitch assumed also that there was a general-purpose system, referred to as the Central Executive, that supported control processes (e.g., reasoning and language comprehension). The articulatory or phonological loop was found to be disrupted by phonological similarity (e.g., Baddeley, 1966; Conrad, 1964) and word length (e.g., Baddeley, Thomson, & Buchanan, 1975) of items in a verbal list to be remembered, by presentation of irrelevant speech sequences (Salamé & Baddeley, 1982) and by asking participants to repeat an irrelevant word (articulatory suppression: Murray, 1965), thought to disrupt the process of mentally rehearsing verbal items in the list (see reviews in Baddeley, 1986; Baddeley et al., 2020).

Baddeley and Hitch (1974) also noted the report of a patient, KF (Warrington & Shallice, 1969) who, following brain damage from a head injury, had a selective impairment of verbal working memory, with a verbal span of just two items for auditory presentation, but intact ability to remember details of past events, and a higher verbal span with visual presentation along with evidence of visually based errors. Subsequently, several other patients have been reported with a similar pattern of impaired short-term verbal memory, but intact long-term memory and visual short-term memory, notably patient PV (e.g., Vallar & Baddeley, 1984). Reviews of these and other similar patients are given in Cubelli et al. (2023), Shallice and Papagno (2019), and Vallar and Shallice (1990). This pattern of spared long-term memory and impaired verbal short-term memory complemented the case reports of long-term memory impairments highlighted by Atkinson and Shiffrin (1968). These contrasting patterns of selective short-term and long-term memory impairments support the view that, in the healthy brain, short-term working memory and long-term memory are functionally separate but interact within a healthy cognitive system. However, the observation of an impaired short-term memory coupled with intact long-term memory is not consistent with the Atkinson and Shiffrin view that information flows from sensory input through a short-term working memory system into long-term memory (Shallice & Warrington, 1970).

Like Atkinson and Shiffrin (1968), Baddeley and Hitch (1974) mentioned only briefly the possibility that visual and auditory short-term storage might rely on different systems. A range of other studies generated evidence for the concept of visual and spatial short-term memory (e.g., Baddeley, Grant, et al., 1975; Baddeley & Lieberman, 1980; Broadbent & Broadbent, 1981; Brooks, 1968; Phillips, 1983; Phillips & Christie, 1977), on the basis of which Baddeley (1986) proposed the idea of a visuo-spatial temporary store, referred to as the visuo-spatial sketchpad^1^ (VSSP). This was viewed as a system that supports visual mental imagery as well as temporary retention of a small amount of visual information. For a more detailed discussion of the influence of Atkinson and Shiffrin (1968) on Baddeley and Hitch (1974) and subsequent versions of the multiple component framework for working memory see Baddeley et al. (2019).

**Figure 1.**
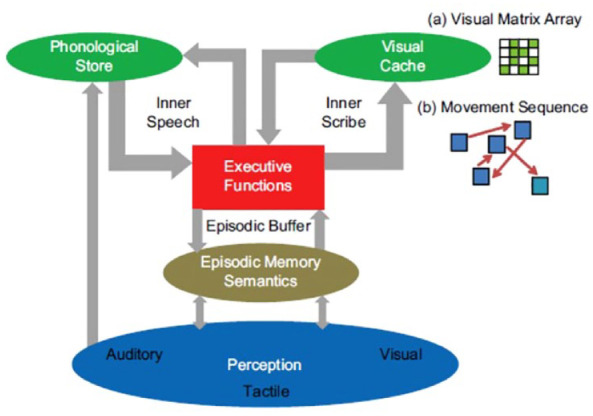
A conceptual view of working memory. See text for a detailed description. *Source.* Reproduced from Logie (2011).

My own PhD research had been on visual temporary storage and processing when participants made mental size comparisons between pairs of named objects and animals (Logie, 1981). While working subsequently with Baddeley on other projects, I pursued this interest in visual working memory, first by showing that not only visual material, but also spatial material could be stored within working memory, and that memory for this material was disrupted by irrelevant non-verbal visual input, but not by irrelevant auditory–verbal input (Logie, 1986; Logie et al., 1990). This was followed by a detailed review of research on visuo-spatial working memory (Logie, 1995) in which I presented evidence for a broad conceptual theory of working memory, and its interaction with stored knowledge, or long-term memory as well as with sensory input. This was based on experimental studies of healthy adults and case studies of individuals with selective brain damage. Rather than develop a whole new theory, I chose to build on previous work and incorporated Baddeley’s ideas for a phonological loop coupled with articulatory rehearsal. This was also broadly in line with the proposal from Atkinson and Shiffrin of an a-v-l store supported by rehearsal, but for temporary maintenance and not for long-term learning. What I added was a more detailed view of visuo-spatial working memory in which mental visual imagery is supported by executive functions, not only by a specialised visual short-term memory system. The latter was proposed to comprise the combination of a passive, non-conscious, limited capacity temporary memory store termed as the *visual cache* that could retain a single visually encoded pattern, and a spatial-based rehearsal system, termed as the *inner scribe* that acted as a control process for retaining movement sequences and spatial sequences but also for rehearsing and maintaining the contents of the *visual cache.* Phillips and Christie (1977) had previously demonstrated a single-item recency effect for the final item in a list of simple matrix patterns. This pointed to a short-term visual memory system that could store a single visual pattern. A slightly modified version of the conceptual theory (Logie, 2011) is illustrated in Figure 1.

As shown in Figure 1, a key aspect of the proposal concerned the flow of sensory input from the environment. In Logie (1995), and as argued by Shallice and Warrington (1970), I noted that, although the evidence of specific verbal short-term memory impairments was consistent with the Atkinson and Shiffrin (1968) separation of short-term working memory from long-term memory, the neuropsychological evidence was not consistent with the proposal that sensory input is channelled through short-term working memory before being transferred to long-term memory. On this view, if short-term memory was impaired, there should also have been problems in accessing long-term memory. Yet long-term memory function appeared to be intact for individuals with verbal short-term memory impairments (Vallar & Shallice, 1990).

**Figure 2.**
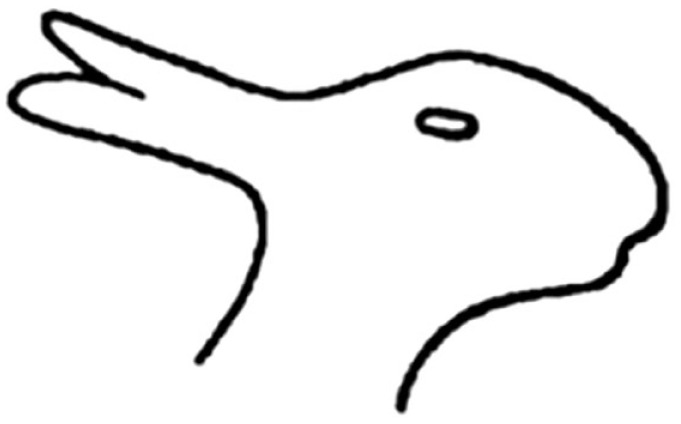
Ambiguous duck-rabbit figure from Chambers and Reisberg (1985).

In Logie (1995), I argued further that the contents of working memory are not raw sensory representations. For example, as noted by Atkinson and Shiffrin (1968), visually presented words are often stored as temporary phonological representations. This would require access to knowledge of the phonology associated with visually presented letters, words, or numbers, *before* being available as a temporary representation in working memory. Moreover, aurally presented words that are names of objects can generate visual mental representations of those objects (e.g., Paivio, 1971). Therefore, any sensory input must first activate relevant stored knowledge before representations are formed in working memory. Further evidence comes from a study by Chambers and Reisberg (1985, 1992), who demonstrated that when the ambiguous duck-rabbit figure (see Figure 2) was shown to each participant very briefly (2 s) and removed, they reported that they saw a duck, or that they saw a rabbit, and they could not change their interpretation based on their mental representation alone. Only when they were later again shown the depicted figure did they notice the alternative interpretation of the figure based on visual perception. This demonstrated that the mental representation is associated with a semantic interpretation that has been generated from activation of stored knowledge, not an uninterpreted sensory image, and is different from viewing a picture. A detailed discussion of this issue is given in Cornoldi et al. (1996). These observations are also not consistent with the Atkinson and Shiffrin proposal that sensory input goes through short-term working memory before it is transferred to long-term memory. These arguments and evidence from studies of healthy adults and neuropsychological case studies led to the depiction in Figure 1, of sensory input first activating stored knowledge in long-term memory and then the products of that activation being transferred to working memory.

Control processes were initially consigned to Baddeley’s (1986) collective, the Central Executive, which Baddeley (1996) described as a “ragbag” of cognitive functions that had yet to be understood. However, the label *Central Executive* raises the conceptual spectre of an unexplained homunculus controlling cognition, with an infinite number of ever higher-level homunculi, each controlling the one below. Atkinson and Shiffrin suggested that there are several different control processes but, like Baddeley and Hitch (1974), did not specify these in any detail. Miyake et al. (2000) identified four such processes in working memory, namely switching, updating, inhibition, and dual task coordination, providing evidence for the first three of these. As a result of concerns about an implied infinity of homunculi, Logie (2003, 2011) referred to a range of “executive functions” (Figure 1). Consistent with Atkinson and Shiffrin (1968), Logie (2003, 2011, 2016, 2018a; Logie, Belletier, & Doherty, 2021) argued that these executive functions can be selectively deployed according to current task demands, and in different combinations by different participants, suggesting that the concept of a single Central Executive, with its implication of a hierarchy of homunculi, had been useful previously but in the light of subsequent findings and insights could now be replaced. A range of studies has demonstrated further that participants may use different combinations of cognitive functions for the same task on different trials or under different experimental conditions (e.g., Bailey et al., 2009; Belletier et al., 2023; Brébion et al., 1997; Brown et al., 2006; Dunlosky & Kane, 2007; Logie et al., 1996; Morrison et al., 2016; Siegler, 1987). A more detailed discussion of the impact of individual differences in cognitive strategies follows in the next section.

Since the early work by Baddeley and Hitch (1974; Baddeley, 1986), the interest in working memory has expanded substantially, but so too has the number of different theoretical perspectives. An influential edited book by Miyake and Shah (1999) comprised chapters from 12 different working memory research groups, and each chapter presented a different theoretical perspective. A more recent edited book by Logie et al. (2021b) comprised chapters from 13 different research groups, 9 of which had developed their own theoretical views on working memory since 2000, so only 4 of these were included in Miyake and Shah (1999). Cowan (2017) described 9 different “faces” of working memory, only 4 of which were represented among the 13 contrasting views in Logie et al. (2021b). Whereas the development of theory can be seen as progress in scientific thinking and understanding, the tendency has been for multiple individual researchers or research groups each to develop a new theory from first principles rather than build on and perhaps improve previous theoretical developments. As a result, some of the research effort has gone into showing how alternative theories are completely wrong, so have little value and should be abandoned in favour of the new alternative. This seems to suggest scientific fragmentation rather than genuine scientific progress towards a common understanding.

Although there are now many different theoretical frameworks for working memory that vary in their detailed assumptions and predictions, often they are characterised under two broad headings. One of these is as described above, comprising a range of continuously interacting, but distinct components that function separately from, but also interact with long-term memory. The other views working memory, not as a separate system, but as the currently activated contents of long-term memory coupled with a limited capacity focus of attention (e.g., Cowan, 1995, 1999, 2005/2016; Cowan et al., 2021). Recent developments have suggested that the differences between these broad approaches might be more apparent than real, reflecting different levels of explanation rather than mutually incompatible theoretical perspectives. I will return to this issue in more detail later when discussing the approach of attempting to resolve debates through adversarial collaboration.

## Conclusions from aggregate data may be misleading

In his book *Remembering*, Bartlett (1932/1961) was concerned that the understanding of psychology could be obscured by focusing on aggregate data across groups of participants, thereby ignoring individual differences between those participants in how they performed the tasks he set. As a result, on page 9 he stated that: “In this book there will be no statistics whatsoever.” Instead he relied on demonstrations of findings that replicated across multiple individual participants, and not on summary data from groups.

Bartlett was not the first to raise concerns about the use of aggregate data across participants. Some 78 years earlier, Bushnan (1854) was equally sceptical:

(statistics) . . . is rather an art than a science; and when unskilfully practiced, is subject to the greatest possible fallacies. The evidence of statistics ranges through the possible, the probable, and the certain. Hence the errors, as the evidence of statistics is assumed to possess one uniform demonstrative character. (p. 18)

Bartlett was quick to state that statistics is a powerful tool for research in psychology, but has to be considered as only one of a range of tools to aid scientific understanding, and not to be used to support the assumption that all participants perform any given experimental task in the same way. This contrasts with the widespread approach of drawing conclusions from comparing distributions of aggregate data from multiple participants across experimental conditions or across participant groups. This has been a dominant approach throughout the intervening years, and is still widely adopted, including in much, but not all, of my own work. The general assumption is that variation in data patterns from individuals is largely due to random noise, possibly derived from differential engagement, motivation and attention by participants, or differences in ability to perform the task. By examining the aggregate data, this random variation should cancel out across participants, and reveal a meaningful consistency that can be attributed to experimental manipulations. The emerging averaged consistency is then assumed to reflect a general principle regarding the impact of a given set of experimental manipulations on performance, and to reveal something of the cognitive functions that support performance.

However, this approach does not consider the possibility that each task might be performed in different ways by different participants, and not only that participants vary in overall levels of performance. The underlying cognitive processes involved for any given task might differ between participants, with subgroups of participants using alternative strategies to meet task requirements, even if the majority of participants perform the task in a similar way. Therefore, the aggregate pattern across the group could be very misleading if it is assumed to reflect a general principle of memory. That pattern might reflect just one of several ways in which the task can be performed, or might even be an artefact of averaging and not reflect the cognition of any individual in the group. Moreover, if different strategies can generate very similar levels of performance on a task, then a high correlation between group scores on different tasks does not necessarily indicate an overlap in the use of the same underlying cognitive function. One reason for this difficulty could be that the focus in many experiments is on building theories of task performance or of observed effects (e.g., Oberauer et al., 2018; for commentaries see Logie, 2018b; Vandierendonck, 2018). It is important to explore effects that arise consistently from multiple experiments, but it is also important to consider that those effects might be generated in different ways (for a related argument see van Rooij & Baggio, 2021). Specifically, the approach is to ask what are the emerging general principles arising out of the aggregate data when participants perform a particular task under specific experimental conditions. I would argue that a more appropriate focus should not be on developing theories based on tasks or effects, but rather should be understanding the organisation of cognition and how it can be, and is deployed flexibly, and in different ways by different people to meet task requirements in the experimental psychology laboratory and in everyday life. In other words, what are the different ways in which tasks can be performed or effects generated?

The concern that aggregate data might not reflect data from individual participants was raised some years after Bartlett (1932), by Sidman (1952) and Estes (1956) in relation to changes in patterns of performance across learning trials. Later still, Siegler (1987) demonstrated the problem by documenting different strategies among children performing mental addition, and suggested that debates among cognitive models of mental arithmetic might simply reflect different ways in which participants performed the tasks in different studies, or differences across laboratories in the methods and measures used. That is, several models of mental addition could be correct, depending on how mental addition was accomplished by the participants being tested. The key issue is to understand what cognitive functions might be deployed to perform mental addition, and how individual participants vary in the cognitive functions they bring to bear on the task, rather than attempt to develop a cognitive theory of the addition task. This points to an approach that considers the patterns of data from multiple individual participants rather than relying on group aggregate data.

The approach of considering multiple individual participants has been common in neuropsychological studies (for recent reviews see MacPherson & Della Sala, 2019; Shallice & Papagno, 2019). Caramazza (1986; Caramazza & McCloskey, 1988) argued that brain damaged individuals are heterogeneous in the type and location of the lesion, the extent of the damage, in the cognitive and behavioural impairments observed, and in any strategies that they develop to cope with those impairments. Indeed, programmes of neurorehabilitation typically are based on the needs of the individual patient and often involve helping them to develop strategies to perform everyday tasks. One approach is to develop the use of cognitive functions that remain intact to perform tasks that previously used cognitive functions that were impaired by the brain damage (for a recent review see Wilson, 2023). Therefore, it is inappropriate to treat such individuals as a group for extracting general principles. Caramazza (1986) proposed that a more robust and rigorous approach would be to undertake extensive cognitive testing of multiple individual cases, and to consider similarities and differences across cases in the patterns of impairment and sparing of cognitive function. This would provide greater understanding of the cognitive challenges faced by each individual: what they can or cannot do in their daily lives. Extending this argument, healthy participants might also be rather more heterogeneous in how they perform tasks than has been widely assumed, even if that heterogeneity is not necessarily as extreme as is found across different forms of brain damage. In the following section, I will consider how investigation of heterogeneity in the cognitive strategies adopted by participants can yield new insights, and help resolve a wide range of scientific debates regarding the understanding of cognition in the healthy brain.

## Strategies in working memory tasks

Dunlosky and Kane (2007) reported evidence for variation in strategy use for what they refer to as “Operation Span,” a version of what is sometimes called Working Memory Span or Complex Span (e.g., Conway et al., 2005). In the Operation Span task, participants are presented with a series of simple arithmetic sums for participants to verify, and each sum is followed by a word that the participants should read and try to remember. After a series of between two and six sum–word pairs, participants are asked to recall the words in the order in which they were presented. Dunlosky and Kane observed a variability in the strategies that participants adopted for task performance, and found that use of an optimal strategy was closely related to overall task performance. This is consistent with findings from a range of other studies, including those in which participants used or were instructed to use specific strategies (e.g., Gonthier & Thomassin, 2015; McNamara & Scott, 2001; Turley-Ames & Whitfield, 2003).

For the present discussion, it is clear that participants may attempt working memory span tasks in a range of different ways, and variation in working memory span scores might reflect the choice of strategy. Further variations in strategy use for working memory tasks were reported by Morrison et al. (2016) and Gonthier (2021). These findings suggest that although the capacity of working memory clearly is limited, measures of working memory span might not provide a precise measure of the working memory capacity of any given participant. Rather, these measures might reflect the strategy that participants adopt for task performance, how efficiently they can use their chosen strategy, or indeed the extent to which they can develop new strategies that enhance their span.

Dunlosky and Kane used the concept of “algorithmic hypotheses” to refer to the interpretation of data in terms of cognitive strategies that participants might adopt for task performance, and “architectural hypotheses” to refer to the organisation of the cognitive system that can support the use of different strategies. This distinction is compatible with the argument that healthy human adults have a range of cognitive functions available and they deploy these functions selectively and in different combinations to meet the requirements of tasks that they have to perform in everyday life as well as in the experimental psychology laboratory.

The same issue arose from a different direction in data from healthy participants, who were acting as neurotypical controls in studies of brain-damaged individuals with short-term verbal memory impairments but normal speech production, and individuals suffering from anarthria (inability to speak). Individuals with impairments of verbal short-term memory typically do not show the effects of phonological similarity or of word length in ordered recall of word lists that have been shown repeatedly in group studies of healthy participants (see earlier discussion in this article and reviews in Cubelli et al., 2023; Shallice & Papagno, 2019; Vallar & Shallice, 1990). However, it appears that the ability to speak is not essential for the functioning of verbal short-term memory. Several studies of individuals with anarthria have reported clear evidence of both phonological similarity and word length effects when such patients were tested by written or typed recall, or serial order recognition (e.g., Baddeley & Wilson, 1985; Bishop & Robson, 1989; Della Sala et al., 1991; Vallar & Cappa, 1987). In a completely unexpected result, when Della Sala et al. (1991) tested a group of 15 healthy controls that were matched on age and education with an anarthric individual LB, they noted that five of the healthy individuals did not show reliable phonological similarity and word length effects, although the effects were statistically significant in the aggregate data across all participants. This was in striking contrast with the presence of these effects in individuals with impairments of speech, including LB. Della Sala et al. (1991) followed up on the healthy individuals who failed to show the standard effects, yet had overall performance levels that were similar to the group means. It appeared that these participants had been using mnemonic strategies to remember the word sequences, and so were not reliant on phonological codes.

These unexpected findings of a lack of standard verbal short-term memory effects in a sample of healthy adults led to a follow-up study by Logie et al. (1996), with a much larger sample of 252 healthy volunteers from the general public. As with the earlier study, in the group aggregate data there were statistically significant effects of phonological similarity and word length with both visual and auditory presentation, and written, serial-ordered recall, but 43% of the participants did not show all of the effects reliably. We asked participants to report any strategies that they had adopted while doing each task, and found that the sizes of the phonological similarity and word length effects were closely related to whether or not participants reported reliably using subvocal rehearsal to remember the word sequences. Participants reporting the use of visual mnemonics and semantic strategies did not show the effects. In other words, the proposal of a phonological store coupled with a subvocal rehearsal mechanism only works as an explanation for immediate, serial ordered verbal recall if participants are using phonological codes and subvocal rehearsal to perform the task.

Further evidence for the diversity of strategies in immediate serial ordered recall came from early brain imaging studies in which participants were free to perform the task as they wished. Across several studies and across participants, a broad range of brain areas was found to be activated, in particular bilateral supplementary motor area (SMA), Broca’s area, mid-dorsolateral frontal cortex, left posterior parietal cortex, left supramarginal gyrus, and the cerebellum (e.g., Awh et al., 1996; Paulesu et al., 1993; Smith et al., 1998). In a subsequent study, Logie et al. (2003) instructed participants to use subvocal rehearsal to retain, and orally recall visually presented lists of five letters (consonants) that were either in random order (e.g., B-Z-R-F-Q), requiring temporary retention of serial order, or in alphabetical order where serial order was well learned (A-B-C-D-E). Retention of serial order compared with rehearsal of the learned sequence was associated with activation in a restricted range of brain areas, specifically the left inferior parietal gyrus and the inferior and middle frontal gyri. Similar findings have been reported since, and recently by Guidali et al. (2019).

Majerus (2019) reviewed a wide range of behavioural and neuroimaging evidence demonstrating the use of multiple processes that are used simultaneously to support immediate memory for serial order when participants were free to adopt their own strategies to support performance. In sum, the phonological loop concept might reflect one of a range of components of cognition that participants can use to support serial ordered verbal recall, but on its own, it does not offer an adequate basis for understanding the range of cognitive functions that participants can deploy to perform verbal serial ordered recall tasks.

### Verbal serial ordered recall

A major strength of the phonological loop concept is that it has been shown to play an important role in retaining serial order of sound sequences within words when acquiring new vocabulary, is closely associated with the development of general language abilities in children (e.g., Gathercole, 1995; Gathercole & Baddeley, 1989; Gathercole & Baddeley, 1993), and has been shown to be involved in counting and mental arithmetic (e.g. Hitch, 1978; Logie & Baddeley, 1987; Logie et al., 1994). Also, across multiple studies (e.g., Tree & Playfoot, 2019; Vallar, 2019; Valler & Baddeley, 1984; recent review in Cubelli et al., 2023), it has been shown to offer insight into the characteristics of verbal short-term memory impairments following brain damage.

One major limitation of the phonological loop concept is that it specifies the use of phonological codes, but does not specify how the retention of serial order of those codes is achieved. In a comprehensive review of studies and computational models of verbal serial ordered recall Hurlstone et al. (2014) focused on phonologically based encoding of the to-be-remembered materials, and commented that there are ongoing debates about which of a range of computational models best fits the data from performance of serial ordered recall tasks. In Logie (2018a), I briefly reviewed these competing models, and argued that each could accurately reflect a different way of remembering a sequence and performing immediate serial ordered verbal recall. For example, one early proposal was that verbal items are retained as a chain of associations, with the recall of the first item acting as a cue to recall the second item, and so on, throughout the list (Ebbinghaus, 1885/1964; Lewandowsky & Murdock, 1989). However, this does not work for lists that are too long for completely accurate recall and result in errors. In a list of eight items, if Item 4 cannot be recalled, then there is no cue available for Item 5 onwards. As a result, the whole of the remainder of the list should also be forgotten. However, typically, people can recall items following a forgotten item. Moreover, some errors involve transpositions, for example, in which Item 5 is recalled before Item 4 of a longer list, and it is not uncommon for the final one or two items of a list to be recalled more accurately than items from the middle of the list, with items at the beginning of the list remembered best of all. These patterns of errors are not consistent with a chaining hypothesis.

One alternative is that the order of items is encoded in terms of the strength of activation of each item in memory, and items are recalled according to their level of activation (Page & Norris, 1998). According to this view, Item 1 is the most highly activated and is recalled first, Item 2 is the next most highly active and is recalled second, and so on. Items that are forgotten do not influence recall of items later in the list, and transpositions occur because items in the middle of the list have very similar levels of activation. A third alternative is that there is a separate learned representation of serial order, and so Item 1 is associated with an external representation of the first list position, Item 2 with the second list position, and so on (e.g., Burgess & Hitch, 1999; for recent discussions see Hitch, 2023; Hurlstone & Hitch, 2018). Again, forgetting Item 4 or 5 would not affect recall of later list items, and items in very similar middle list positions could be swapped at recall. In principle, items could be recalled with list position as a cue: for example, recall Item 7, now Item 2, now Item 5, and so on, and not only in serial order.

As argued in Logie (2018a; for a related argument see Logan & Cox, 2021), each of these views could reflect a different strategy for retaining serial order, and could also be influenced by the length of the list. For example, to learn a new word in our own or in a new language, it is essential to remember and reproduce the sequence of sounds in the word. To learn the Japanese word “arigato” meaning “thank you,” it is important to remember the sequence ar-i-ga-to in the correct order, and most words in most languages comprise one, two, three, or four syllables. For most healthy adults, errors would be very rare in recall of a sequence of four or fewer items, and a chaining of associations between the word sounds could work rather well to retain and reproduce serial order. In contrast, learning the German word “arbeiterunfallversicherungsgesetz,” meaning “worker accident insurance act,” is rather longer and more likely to be prone to error in recall by someone learning German for the first time. In this case, a chain of associations strategy might not work quite so well, and it might require reference to some learned representation of serial order that encodes “ar” in Position 1, “beit” in Position 2, and so on. In this case, there could be a transposition error with perhaps “fall” and “ver” swapping places to generate “arbeiterunverfallsicherungsgesetz.” For even longer lists, perhaps of 12 or more items, neither an association chain nor positional encoding would work too well, and so using different levels of activation across the sequence of items might be a better strategy. Some support for this view comes from a study by Ward et al. (2010), who showed that for longer lists, people tend to recall items in any order they can remember them (free recall) even if they are instructed to remember in serial order. That would be consistent with recall based on the levels of activation of each item. For shorter lists, perhaps six or seven items, people tend to recall them in serial order even if they are instructed to use free recall. This would be more consistent with the use of links with list position. In other words, all three models for retaining serial order could offer a match with a different strategy, depending on how many items are to be retained and how participants choose to perform the task. As argued by Majerus (2019), more than one strategy might be adopted simultaneously, for example, using both item links and different levels of activation across the list.

In addition to serving as an example of how different strategies might be used to perform the same task (serial ordered recall), this general topic area also offers a useful example of researchers who are associated with different computational models interacting with a view to seeking a common solution, rather than working in isolation (for a recent review see Hitch, 2023).

### Visual coding in verbal serial recall

**Figure 3.**
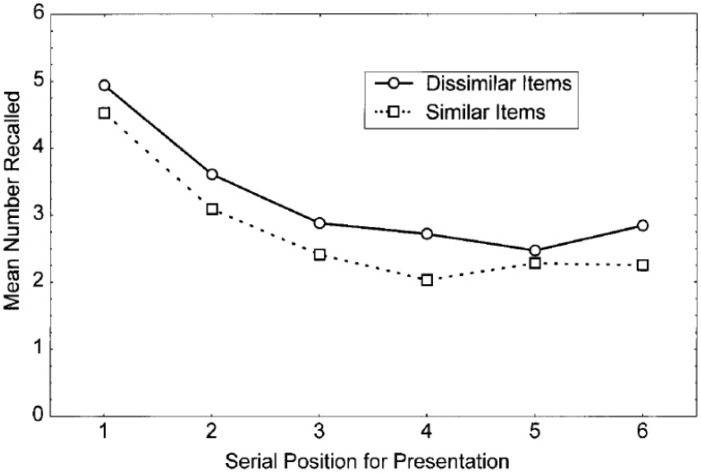
Mean correct serial recall for visually presented six item lists. *Source.* Reproduced from Logie et al., (2000).

In the Hurlstone et al. (2014) review, it was stated that there was very limited research on visually based serial ordered recall. However, in addition to the general evidence for variation in strategies for performing immediate verbal serial recall, there was evidence that participants may encode the visual appearance of visually presented verbal lists in addition to the phonological characteristics. Logie et al. (2000) selected sets of words that are visually as well as phonologically similar (*Fly, Ply, Cry, Dry, Shy*), and contrasted these with words that are phonologically similar but visually dissimilar (*Guy, Thai, Sigh, Lie, Pi, Rye*). In addition, for half of the trials, participants had to repeat aloud the irrelevant word “hiya” (articulatory suppression) throughout presentation and written recall of the visually presented lists. As illustrated in Figure 3, when controlling for phonological similarity, words that were visually similar were recalled less well than words that were visually dissimilar. The effect of visual similarity was shown by 15 of the 16 participants, suggesting that nearly all participants were using visual codes to help support their retention of the word sequences. Articulatory suppression reduced overall performance for all 16 participants, but this did not interact with visual similarity, which also appeared for all 16 participants, suggesting that all participants might have used visual codes to help them remember the ordered sequence when suppressing articulation and trying to remember phonologically similar words. The finding of a visual similarity effect was replicated in a second experiment with different sets of words.

A further two experiments in Logie et al. (2000) presented sequences of four letters shown in a mixture of upper and lower case, drawn from contrasting sets of six letters. For one set of letters the upper and lower case versions look very similar, Kk Ww Yy Cc Xx Zz. For the other set, the upper and lower case versions look very different, Hh Ll Rr Qq Dd. Participants were asked to recall the order in which the letters appeared, but also the case in which each letter appeared (e.g., KzwC or hRqD), without and with articulatory suppression. Recall accuracy was poorer for the letters in which upper and lower cases are visually similar, shown by 14 of the 20 participants, with three of the participants performing at ceiling. Three other participants did not show the effect. However, the disadvantage for the visually similar letters was greater under articulatory suppression, with all 20 participants showing an effect of visual similarity as well as a reduction in overall performance. The latter result suggested that both phonological and visual codes were being used, possibly with greater reliance on phonological codes when there was no articulatory suppression and an increased reliance on visual codes when articulatory suppression made articulatory rehearsal more difficult. That is, at least some participants appeared to change their encoding strategy for verbal serial recall between the conditions without and with articulatory suppression. A similar pattern of results was found in Experiment 4 with different sets of letters.

These findings were replicated in later studies with Japanese participants, who were presented with sequences of Japanese kanji characters that varied in visual as well as phonological similarity (Logie et al., 2016; Saito et al., 2008), and provided evidence that both phonological and visual codes were being used at the same time to support written recall performance of each list.

Several other studies have reported evidence for the use of visual codes in immediate memory for verbal material, using a range of different paradigms with English language materials (e.g., Parks et al., 1972; Posner et al., 1969; Posner & Keele, 1967; Wolford & Hollingsworth, 1974) and with Chinese (Hue & Ericsson, 1988; Lin et al., 2015; Yik, 1978). Manning (1977) noted that letters in the English language are inherently more distinct visually than they are acoustically. Therefore, acoustic or phonological confusions are much more likely to arise spontaneously than visual confusions with English language materials, unless the materials are selected specifically to manipulate visual similarity. The results provided evidence for a cognitive function that can support visual coding and that may be deployed strategically to support verbal serial ordered recall. That is, the data point to development of a theory of the range of cognitive functions available to support performance of a wide range of tasks, and not to a theory of the task of verbal serial ordered recall.

More recently, Baddeley et al. (2023) found evidence of phonological similarity in recall of Chinese characters by Chinese native speakers, and surprisingly this effect was only slightly disrupted by articulatory suppression, in contrast with the impact of articulatory suppression on recall of English language materials (Murray, 1965). This suggested that the Chinese speakers could retain phonological codes in a form that is not sensitive to this form of disruption that typically removes the phonological similarity effect with English language materials (Baddeley et al., 1984). They also found no evidence for the use of visual codes, although this was not a major focus of the study. This suggests that there is yet another phonologically based encoding strategy being used by their participants, although further studies would be required to identify precisely what that strategy might be, what kind of cognitive function might support it, or indeed if several different strategies are being used across participants.

### Strategy variation for non-verbal visual tasks

**Figure 4.**
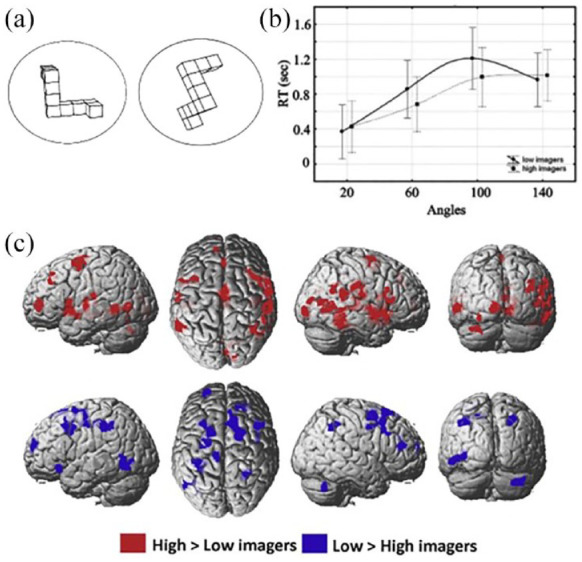
(a) Example stimuli for mental rotation. (b) Response times for different angles of rotation for high imagers and low imagers. (c) fMRI contrast images between high imagers and low imagers performing mental rotation. *Source.* Reproduced from Logie et al. (2011).

Zacks (2008) carried out a meta-analysis of a wide range of studies that had used brain imaging techniques, notably functional magnetic brain imaging (functional magnetic resonance imaging [fMRI]), to investigate the brain areas that are associated with performance of a widely used task referred to as mental rotation. This involves presenting two very similar three-dimensional objects depicted in different orientations (see Figure 4a). Originally developed by Shepard and Metzler (1971), the task involves deciding whether the two depicted objects are the same or different. Behavioural results across a very large number of studies have shown consistently that the larger the difference in the angle of orientation between the depicted objects, the longer participants take to make their decision. The most widely held assumption is that participants mentally rotate a visual image of the objects so that they are imagined in the same orientation as they would if the objects were physically present. The larger the angle, the longer it takes for the mental rotation. The intriguing conclusion from the Zacks (2008) review of brain imaging studies on this task was that, across studies, nearly every area of the brain had been reported as being involved. This suggested that there was a dramatic lack of reliability and replicability across brain imaging studies, and/or that participants might be doing this task in different ways in the different studies, and so different brain networks were involved in the use of different strategies.

To test the hypothesis of strategy variability in mental rotation, Logie et al. (2011) contrasted the behavioural data and brain activation patterns from participants who rated themselves as high imagers on the vividness of visual imagery questionnaire (VVIQ, Marks, 1973), with the behavioural data and brain activation patterns from participants who rated themselves as having little or no experience of mental visual imagery. As illustrated in Figure 4b, all participants showed the typical behavioural pattern of longer decision times for larger angles of rotation in the depicted object pairs, at least up to a rotation angle of 100°, although the high imagers had faster times overall than the low imagers. The response time data beyond 100° suggest that the low imagery group might have a strategy that is insensitive to large rather than small angles of rotation. In other words, the two groups might be attempting the task in different ways, or that low imagers are just not as efficient at mental rotation as the high imagers, at least for the smaller angles. The fMRI results showed that there was a broad overlap for all participants in the activation of the pre-motor cortex relative to a rest (no mental rotation) condition. However, the fMRI contrasts between the participant groups, shown in Figure 4c, indicated that multiple very different additional areas of the brain were activated during mental rotation, and multiple different areas were activated for the high imagers than for the low imagers and vice versa. This is consistent with the conclusion from the Zacks (2008) meta-analysis that, across brain imaging studies multiple brain areas appear to be involved. Our data suggest that at least some of that variability may be due to how different participants attempt to perform the task.

In a related neuropsychological case study, Zeman et al. (2010) carried out a wide range of tests on an individual, MX, who, following angioplasty for a heart condition, reported a sudden complete loss of the ability to create and manipulate visual images, having previously used visual imagery throughout his working life as a quantity surveyor as well as in his everyday life outside of work. Structural MRI showed minor white matter high intensities and borderline fronto-temporal atrophy, neither clearly falling outside the normal limits for his age, and there was no other evidence of brain damage.

**Figure 5.**
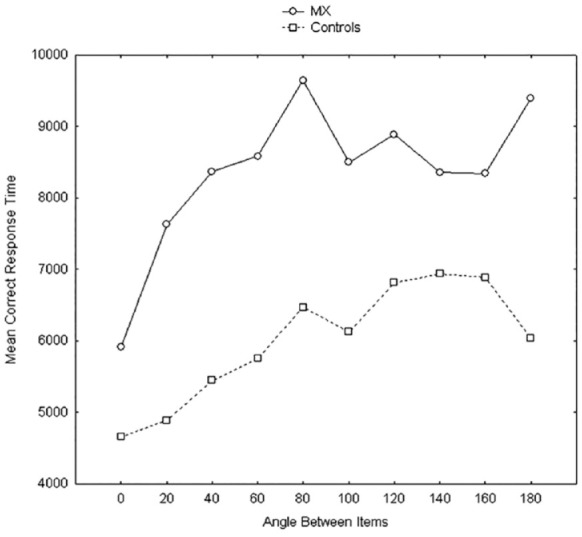
Response times across angle of rotation for case study MX and 10 matched control participants. See text for details. *Source.* Reproduced from Zeman et al., (2010).

He reported the sudden onset of an inability to imagine the face of his wife or close friends, of familiar scenes or objects. When tested, he performed well within the normal range on measures of language, memory, including visual short-term and long-term memory, and was well above average intelligence, although his non-verbal IQ was lower than his verbal IQ scores. He had a low score on the self-report VVIQ, but no objective measures indicated an impairment. This conclusion changed when he was tested on mental rotation. His data and the average data from 10 control participants matched with MX on age and occupation, as shown in Figure 5. In line with many previous studies, the results were consistent across the 10 control participants in showing the typical increase in response time with increasing angle between the depicted objects,^2^ but a shorter response time at 180° which is effectively a mirror image that is easier to reverse than mentally rotate. In contrast, although MX showed an increase in time between 0 and 40°, thereafter, his response time function was essentially flat until 160°, and if anything, shows a further increase for 180°. His overall accuracy was equal to that for the controls. When asked about how he was attempting the task, MX reported visually comparing the angles between objects. Doing so took longer overall, but the strategy resulted in highly accurate performance, and it was insensitive to the angle of orientation between the depicted objects. Following the Zeman et al. (2010) report of MX, Zeman et al. (2015) referred to the loss of, or inability to generate visual imagery as “aphantasia.” Cases similar to MX have been reported more recently by Jacobs et al. (2018), Keogh et al. (2021), and Thorudottir et al. (2020). The separation shown in MX between impaired visual imagery and intact visual short-term memory is consistent with the Logie (1995, 2003, 2011; Borst et al., 2012) proposal that visual short-term memory and visual imagery do not rely on the same cognitive systems, in contrast with what has been and is assumed for the visuo-spatial sketchpad (Baddeley, 1986; Baddeley et al., 2021).

### Implications from strategy variability

The studies discussed in this section all point to the availability and use of different cognitive strategies being used by different participants when performing tasks designed to measure serial ordered recall and mental rotation, including strategies that may be adopted to compensate for an acquired cognitive impairment. In Logie (2018a), I discussed a range of related studies in different research areas that demonstrated selective strategy use across participants, for both serial ordered recall tasks and tasks designed to assess mental visual imagery. In a related argument, Pearson and Kosslyn (2015) suggested heterogeneity of strategies for visual imagery tasks could end the long-running debate about whether visual imagery is functional in cognition or an artefact of conscious experience (e.g., Kosslyn, 1994; Paivio, 1971; Pylyshyn, 1973, 1981). They concluded that imagery is an optional strategy that might or might not be used for performing imagery tasks, and that some people simply do not experience visual imagery. This is consistent with the studies described above regarding MX (Zeman et al. (2010) and differential activation patterns for high imagers and low imagers performing mental rotation (Logie et al., 2011), as well as the Zacks (2008) meta-analysis showing poor consistency across brain imaging studies of mental rotation. Subsequently, Pearson and Keogh (2019) proposed a brain imaging-based approach to studying strategy differences in visual working memory tasks. If accepted by both sides of the debate about whether or not imagery is functional in cognition, this proposed resolution is very welcome. However, consistent with the argument that binary debates can delay scientific advance, it is notable that this proposed resolution based on strategy variability was published after more than 40 years of debate (Paivio, 1971; Pylyshyn, 1973).

My arguments in Logie (2018a) were broader in pointing to evidence that variation in cognitive strategy could account for a wide range of debates across a diversity of research topics, with further examples from studies of domain-specific expertise and age-related changes in cognitive performance. I will not reiterate those arguments in detail, but in summary, there is considerable evidence that people with domain expertise perform tasks in that domain very differently from people without that expertise. Among the numerous domains of expertise that have been explored experimentally are chess, history, mathematics, sport, dance, and ballet, memory for menu orders, digit sequence recall, and mnemonic use (reviews in Ericsson et al., 2018), as well as memory for soccer scores (Morris et al., 1985) and residential burglary (Logie et al., 1992). On the same principle, strategy differences across participants might be viewed, at least in part, as reflecting variation in individual differences in modest levels of expertise with particular mental abilities (e.g., visual imagery, verbal rehearsal, mental arithmetic).

**Figure 6.**
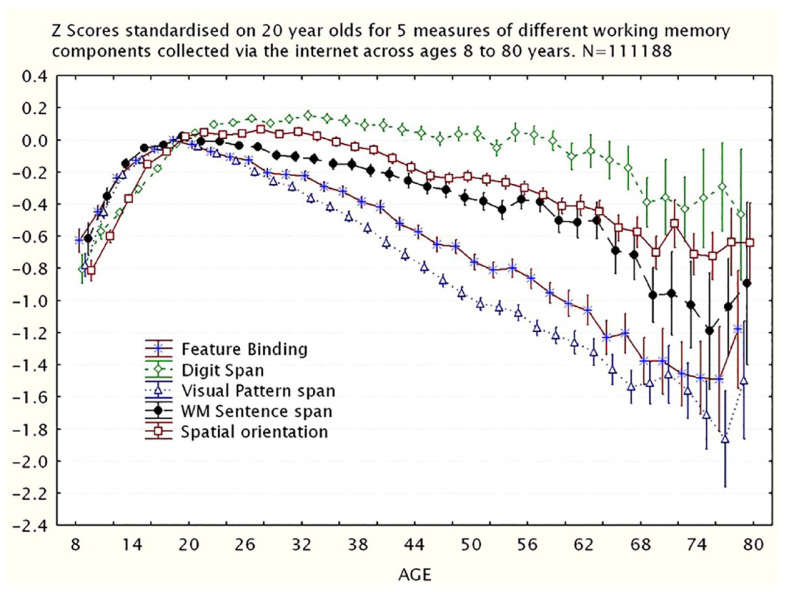
Z Scores standardised on 20-year-olds for five measures of different working memory functions collected via the internet from 111,188 participants aged 8–80 years. *Source.* Reproduced from Logie et al. (2015).

**Figure 7.**
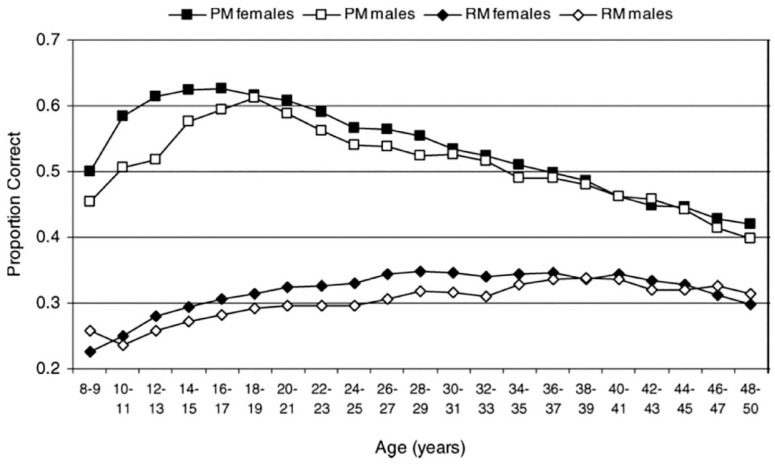
Proportion of correct responses from 318,614 participants aged 8–50 years for one trial assessment of prospective memory (PM) and retrospective memory (RM), plotted in the 2-year age bands, separately for male and female participants. *Source.* Reproduced from Maylor and Logie (2010).

In the case of healthy ageing, there is growing evidence that older people might perform tasks differently from younger people, and so there could be age-related changes in cognition that do not result in age-related decline in performance (e.g., Logie et al., 2015; Perfect & Maylor, 2000). Age-based contrasts in performance may reflect strategies developed from life experience and acquired knowledge that also can compensate for general age-related cognitive decline (e.g., Baltes & Baltes, 1990; Deary et al., 2012; Forsberg et al., 2020; Johnson et al., 2010; Park & Reuter-Lorenz, 2009 ; Salthouse, 2019). Moreover, strategies developed through life experience might be more or less effective for different kinds of tasks. For example, as shown in Figure 6, from a sample of over 111,000 people between the ages of 8 and 80 years (from a total sample of over 400,000), Logie et al. (2015, 2020; Johnson et al., 2010; Logie & Maylor, 2009; Maylor & Logie, 2010) demonstrated strikingly different age-related trajectories across a range of different cognitive tasks, with, for example, verbal memory span showing no decline until nearly 70 years of age, but a test of short-term visual memory showing significant decline by age 25 years. On a subsample of over 95,000 from this same dataset, Johnson et al. (2010) demonstrated that older and younger people appeared to be performing these two types of tasks in different ways. That is, older people appeared to perform tasks differently from younger people, and the observed task scores suggested the use of very different cognitive functions across age groups for the same tasks. One possibility is that older people tried to use verbal strategies to perform what are designed to be visual memory tasks (e.g., Forsberg et al., 2020). From that same large dataset, Figure 7 (Maylor & Logie, 2010) shows that measures of prospective and retrospective memory have very different age-related trajectories. Therefore, it might not be reasonable to assume that all cognitive tasks decline with age or decline at the same rate, or that any given task is measuring the same psychological construct in younger and older people.

I will return to further discussion of the importance of variation in strategy use in the following section on attempting to resolve long-running theoretical debates about the organisation and function of working memory.

## Debates may inhibit advances in scientific understanding

Advances in scientific understanding, and in particular the understanding of human memory can progress through rare, major findings and insights, or more frequently by slow and systematic accumulation of empirical evidence. The former often take some considerable time to gain independent verification and broad acceptance in the face of the theoretical *zeitgeist*. Interpretation of the latter stimulates scientific debate that is often fuelled by contrasting theoretical frameworks. For example, five decades after both Atkinson and Shiffrin (1968) and Baddeley and Hitch (1974), and a large volume of subsequent studies (e.g., Baddeley et al., 2021) demonstrated separate, but interacting cognitive systems for short-term and long-term memory, there is an ongoing debate about whether they are truly distinct, or that working memory and long-term memory are essentially the same system, with the former comprising currently activated information from the latter (e.g., Cowan et al., 2021).

Debate plays a crucial role in revealing flaws in empirical methodology or findings that are difficult to replicate. However, as demonstrated by the longevity of as yet unresolved debates identified by Newell (1973) and in recent books and review articles on working memory (e.g., Cowan, 2017; Logie et al., 2021b), debate can also hamper genuine progress when it is driven by different laboratories using different methodologies and experimental procedures that are set within the context of different theoretical perspectives or involve different scientific disciplines. The hurdles to progress may be exacerbated by the pragmatics of scientific career development, for example, by building a reputation for developing a particular theoretical perspective and generating multiple experiments that support one’s own theory while being inconsistent with alternatives. Rarely do researchers from different theoretical perspectives collaborate in a common research programme, using a common methodology, and over an extended period.

Addressing theoretically driven debates often follows an adversarial process in which different perspectives compete on the assumption that the winner of this competition will offer the most satisfactory or most widely accepted account of a set of observed findings. However, as noted above, all too often this form of adversarial debate self-perpetuates, and rarely is there an outright winner. Newell (1973) discussed the futility of binary oppositions in cognitive psychology, and listed 24 binary oppositions current at the time, several of which reflected decades of ongoing debate. Despite the subsequent generation of large volumes of data, and developments of new methodologies, many of those binary oppositions remain unresolved after 50 additional years of research. Take for example, the debate about whether forgetting of sets of verbal stimuli arises from some form of decay of a memory trace over time, or from new stimuli interfering with or displacing an existing trace. This was a debate in the first half of the 20th century (e.g., McGeoch, 1932) and remains a debate in the contemporary literature on working memory 90 years later, with some arguing for the contribution of both decay and interference (e.g., Barrouillet & Camos, 2021; Barrouillet et al., 2012, 2018; Cowan et al., 2021) and others arguing that there is only interference or displacement by new material (Lewandowsky & Oberauer, 2015; Lin & Oberauer, 2022; Oberauer, 2021; Oberauer & Lin, 2017). I confess to having contributed to proliferation of some of the debates in the area of working memory (e.g., Logie, 1997, 2012, 2018b, 2019; Logie & Della Sala, 2003, 2010).

Three decades after Newell (1973), Kahneman (2003) was even more critical of long-lasting debates and referred to controversy in science as “a waste of effort.” To paraphrase his comments, he argued that scientific point scoring and angry science are absurdly competitive and demeaning, with either side rarely admitting an error or that they had learned anything from the other side of the debate. Rather than participate in this scientific tennis match (my phrase, not Kahneman’s) of target article—critical commentary-rejoinder, he would rather do something else. He advocated the approach of *adversarial collaboration* to avoid endless controversy and debate. This involves the opponents in a debate committing to a collaborative research programme that involves a common, agreed methodology and with contrasting expectations stated in advance. He participated in some adversarial collaborations himself (Ariely et al., 2000; Gilovich et al., 1998; Mellers et al., 2001), and there had been previous attempts using this approach (e.g., Cowan et al., 1997; Latham et al., 1988). However, each of these cases appeared to be ad hoc and one-off collaborations that resulted in single papers with a small amount of empirical work. There was no long-term follow-up, and there was minimal or no change in the contrasting views, with authors interpreting the results from their own perspective in different sections of the final discussion and conclusions in the paper.

There are good reasons why adversarial collaborations are rare and on a small scale. It is likely to be a significant challenge to get researchers who disagree to participate, and it cannot be driven by only one side of the debate: both research groups have to embrace the rationale for the collaboration. Moreover, significant progress in resolving a well-established debate requires an extended programme of work. A debate that has lasted a decade, or more, will not be resolved with the completion of one or two collaborative experiments: a much more ambitious and extended research programme is needed. Ad hoc and small-scale collaborations can be completed with minimal funding and infrastructure support. A larger scale adversarial collaboration requires substantial funding, and requires substantial effort to persuade a funding agency that the project is worth supporting. In the absence of both the willingness of all parties involved and targeted, longer-term research funding, the widespread practice (that Kahneman described as a waste of effort) of competitive theorising, critical commentary, and response with no resolution on the horizon is set to continue.

## An extended adversarial collaboration: the WoMAAC project

**Figure 8.**
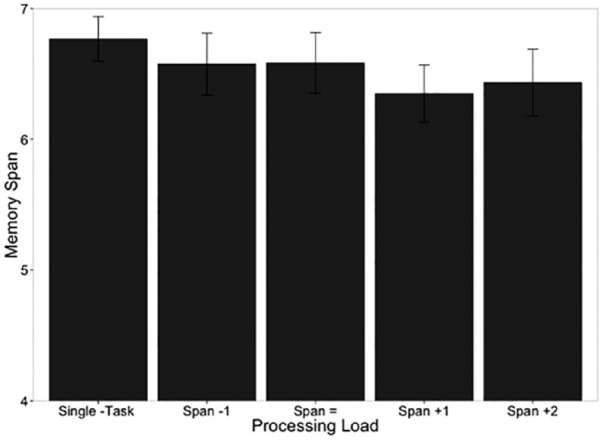
Mean memory span scores (with standard errors) in single task and with a concurrent spatial processing task that varies from below span (span −1) to above span (span +2). *Source.* Reproduced from Doherty and Logie (2016).

**Figure 9.**
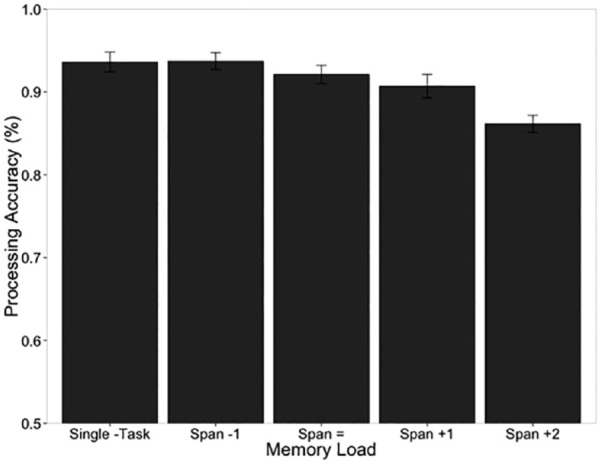
Mean spatial processing accuracy (with standard errors) from single task and with a concurrent memory load varied from below span (span −1) to above span (span +2). *Source.* Reproduced from Doherty and Logie (2016).

I was fortunate to lead an extended adversarial collaboration over a period of 4 years, funded by the UK Economic and Social Research Council, for the WoMAAC project (Working Memory across the Adult lifespan: an Adversarial Collaboration). The regulations for this funding allowed for a proportion of the funds to be subcontracted to researchers in other countries. This allowed for a collaboration between three research teams who each were associated with different and competing theoretical perspectives regarding working memory. The initial conversation regarding this collaboration was triggered by a pattern of data initially presented as a poster (Doherty et al., 2014; published as Doherty & Logie, 2016) at a meeting of the Psychonomic Society. This comprised two dual-task experiments that combined recall of digit sequences with a spatial judgement task during a 6-s retention interval. Crucially, performance was assessed on the basis of the maximum level at which each participant was correct on 80% of trials, that is, memory span and spatial task span for each individual participant for each task. The results were clear in showing that varying the load from below span through to above span for the spatial task during the retention interval did not affect accuracy in recalling the digits, as illustrated in Figure 8. Performance on the spatial task was unaffected by the dual task conditions when both the memory load and the spatial task load were set at the individual participant span levels. Spatial task performance was only affected when the memory load was set above memory span for each participant, as illustrated in Figure 9. These results were consistent with the proposal from multicomponent working memory that short-term verbal memory and the cognitive functions that supported the spatial task (processing) could function in parallel and with no mutual disruption as long as the cognitive load of each task is set within the span or capacity of the cognitive function required to support each task for each participant. Also, consistent with findings from the dual task studies in Baddeley and Hitch (1974), only when the load of one or other task exceeds the capacity of the cognitive functions supporting each task, is there a requirement to share cognitive resources with a consequent impact on overall performance.

These results from (Doherty et al. 2014; Doherty & Logie, 2016) were not consistent with at least one contrasting theory of working memory, time-based resource-sharing (TBRS: e.g., Barrouillet & Camos, 2001; Barrouillet et al., 2004, 2011), in which it was assumed that both memory maintenance and processing require limited capacity attention, so when both memory and processing are required, participants switch attention between them. The more time that is spent with attention focused on processing, the more likely items in memory will be lost over time, and the more time spent with attention focused on retaining the memory items, the less efficient will be the processing. The TBRS framework would predict that performance of the spatial judgement task used by (Doherty et al., 2014; Doherty & Logie, 2016) during a retention interval for a digit sequence should have resulted in poorer subsequent digit recall, which is not what was found. Since it was first proposed by Barrouillet and Camos (2001) the debate with the multiple component approach had, in the main, been following the typical pattern of unresolved debates in cognitive psychology, with each team reporting their own data using their own paradigms with results from each laboratory seemingly contradicting the results of the other laboratory. However, rather than leading to perpetuation of the published debate, the Doherty et al., (2014) poster led to an in-person conversation during a break in the programme for the Psychonomic Society meeting in Long-Beach, California. That conversation between Robert Logie, Pierre Barrouillet, and Valerie Camos led to the idea of conducting an adversarial collaboration to attempt to resolve the published debate.

We decided to invite a third party to join our proposed collaboration, namely Nelson Cowan, who had developed his own theoretical perspective for working memory, namely Embedded Processes (Cowan, 1988, 1995, 1999), the assumptions of which also were not compatible with the (Doherty et al. 2014; Doherty & Logie, 2016) data. In Embedded Processes, working memory comprises the currently activated information from long-term memory that is required for our current task, coupled with a limited capacity focus of attention to a small amount of that information, which can be updated on a moment-to-moment basis. So, performing two tasks was assumed to divide limited capacity attention and lead to poorer dual task than single-task performance. Cowan’s primary motivation was to understand consciousness, and he argued for an explicit link between the focus of attention and the current contents of conscious experience. My own view was, and is, consistent with a statement by Pylyshyn (1973) that what is available in consciousness is not necessarily functional and that not all that is functional is necessarily available to consciousness. For a direct contrast between our views on this issue see Logie and Cowan (2015), the joint writing of which was also an example of adversarial collaboration. Some evidence that not all that is assumed to be within working memory is in consciousness has been reported in studies showing that short-term maintenance during a retention interval can be stored in a passive state without any persistent neural activity (e.g., LaRocque et al., 2013; Rose et al., 2016; Zhang et al., 2022).

There were hints in some of our previous studies that our assumptions were perhaps not as far apart as they might appear, and there was evidence of more than one component of working memory function for both TBRS and Embedded Processes. For example, Camos and Barrouillet (2011) reported evidence of there being two forms of memory maintenance within working memory, noting a developmental change in children from reliance on passive maintenance to using active refreshing of memory contents. Camos et al. (2009) provided evidence that articulatory rehearsal, and active refreshing by focusing attention on the memory items, comprise different maintenance mechanisms. Cowan et al. (2014) demonstrated a dissociation between what he referred to as central and peripheral components of working memory storage, with the latter being not totally dissimilar to the concepts of the phonological loop and visual cache of a multicomponent working memory. There was also theoretical overlap from the multiple component perspective. The role of cognitive functions that support cognitively demanding tasks was incorporated in the original Baddeley (1986) concept of a Central Executive, and as a range of executive functions (Miyake et al., 2000) within my own proposals (Logie, 2011). For example, Logie et al. (1990), Salway and Logie (1995), and Duff and Logie (1999, 2001) all reported evidence of a general dual-task decline in performance as well as task-specific reductions in performance compared with single-task conditions.

I had previously undertaken multiple interdisciplinary collaborations with computer scientists, neurologists, neuropsychologists, neonatologists, criminologists, and a wide range of experimental cognitive psychologists who shared many of my theoretical assumptions. However, this was the first time we had each embarked on an extended collaboration among experimental cognitive psychologists whose theoretical assumptions did not substantially overlap. As far as we are aware, it was also the first adversarial collaboration in experimental cognitive psychology that had extended over several years. The first 6 months of our project were spent discussing and agreeing to our basic experimental paradigm, and our modes of operation. That this extended initial discussion was necessary is another reason why short ad hoc adversarial collaborations are unlikely to lead to a resolution of debates.

Because we intended to include studies of healthy ageing as well as of healthy young adults, one of Nelson Cowan’s colleagues, Moshe Naveh Benjamin joined the team, and our funding allowed us to appoint five, talented post-doctoral researchers. Three of these, Jason Doherty, Agnieszka Jaroslawska (now Agnieszka Graham), and Alicia Forsberg were based with me in Edinburgh, Stephen Rhodes was based with Nelson Cowan and Moshe Naveh-Benjamin in Columbia, Missouri, USA, and Clément Belletier was based with Valerie Camos and Pierre Barrouillet in Fribourg and Geneva, Switzerland.

We were aware that our proposed project represented only three of many theoretical perspectives on working memory, as noted in the “Working memory: development of debates” section of this article. However, we were acutely aware of the need to set up a project that was feasible and that would generate a manageable number of alternative predictions that could be tested empirically. The project was also a test of whether an extended adversarial collaboration is even possible as an approach to directly addressing, and hopefully resolving debates. Having more than three teams of researchers would have led to increased complexities in the logistics of regular communication and agreeing on experimental paradigms. It would also lead to substantial increases in the required costs for the research, making it less likely that a bid for research funding would be successful for what was already a substantial project. Finally, it was important that there was some initial level of agreement between the three teams of researchers, so that we could focus on theoretical differences that were experimentally tractable, that project members were open to the possibility that the experimental results might challenge their theoretical perspectives, and that despite ongoing professional scientific debate, personal interactions would remain friendly.

To ensure a productive and successful project, we first agreed on a number of working practices. The design, procedure, and analysis plan for each of the main 15 experiments were discussed and agreed in advance, and all these details were uploaded to the Open Science Framework along with predictions from each laboratory based on their own theoretical perspective. Identical equipment and software were purchased and used in each of the three laboratories, and each experiment was carried out independently and in parallel in two out of the three laboratories. Experiments with young healthy adults were carried out in Edinburgh and in Fribourg and Geneva. Studies on healthy ageing were carried out in Edinburgh and in Columbia. After data collection, analyses were carried out in parallel on the data from each laboratory, and then the datasets were combined for an overall analysis to detect any differences in data patterns between laboratories. It turned out that between-laboratory differences were minimal and largely restricted to different levels of performance rather than different data patterns. All of the data from each experiment were then also uploaded to the Open Science Framework. For each of the empirical papers from the project, one or other of the post-docs was responsible for drafting the paper and was the lead author. All post-docs and the five co-principal investigators (PIs) were co-authors on each major paper. If any of the post-docs wished to pursue related, additional research, that was encouraged (e.g., Jaroslawska & Rhodes, 2019) as long as it did not interfere with the main research programme. The post-docs were responsible for the day-to-day running of the experimental work, were in almost daily contact electronically, and each had the opportunity to visit and work in one of the partner laboratories, in some cases for several months. Finally, at key points before and after experiments, there were online video meetings between all three teams. Also, there were in-person project meetings at each laboratory, and when team members were attending the annual meeting of the Psychonomic Society.

Most of the WoMAAC experiments involved investigations of the conditions under which performance does or does not decline under dual-task compared with single-task conditions, following the Doherty and Logie (2016) study. An important detail of the procedure was that the load for each experimental task was set at the measured span (i.e., titrated) for each individual participant. As shown in Doherty and Logie (2016), performance was reduced under dual-task conditions only when the demand of each task exceeded the capacity (span) for each participant when performing under single-task conditions. If the demand of each task had been set at the same level for all participants, this could have exceeded the spans of some or even all of the participants. It was acknowledged among WoMAAC project members that at least one possible reason for previous contrasting results between laboratories on dual-task performance was because studies within the multicomponent framework almost invariably titrate task demand for each participant, whereas this was not typically the case for the other laboratories. For example, a wide range of studies have reported that older participants show a greater reduction in dual-task compared with single-task performance than do younger participants, known as an age-related dual-task cost (e.g., review in Kilb & Naveh-Benjamin, 2015). However, in a meta-analysis completed within the WoMAAC project, Jaroslawska and Rhodes (2019) reported that in studies for which single-task performance was titrated, there was little or no evidence for an age-related dual-task cost, consistent with several other reports (e.g., Salthouse, 2015, 2019).

During the planning and design of each experiment, each of the three research teams generated predictions for the experimental outcomes based on their theoretical perspective.

**Figure 10.**
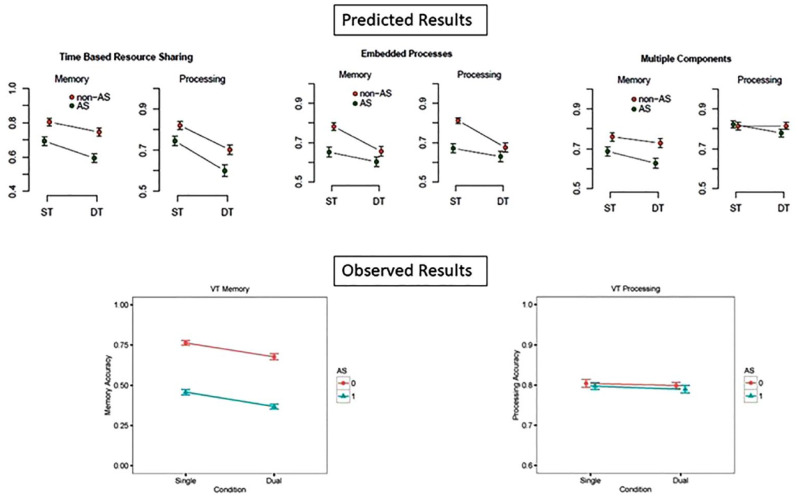
Predicted results from three theoretical perspectives, and observed results for single-task and dual-task serial recall of random letter sequences (memory) and simple arithmetic verification (processing), each set at the span for individual participants. Tasks were performed without and with articulatory suppression. Data are reported in Doherty et al. (2019). Figure is drawn for the current article.

The details of these experiments have been reported elsewhere (e.g., Doherty et al., 2019; Jaroslawska et al., 2021; Rhodes et al., 2019, 2021; for a comprehensive list see http://womaac.psy.ed.ac.uk/research-output). As an illustration, Figure 10 shows the predictions from each theoretical perspective for one of the experiments reported in Doherty et al. (2019) along with the observed results. Participants were asked to retain a random sequence of letters (at their measured span length) for the memory task, and to undertake simple arithmetic verification (e.g., 7 + 4 = 12, true or false) as a processing task during a 10-s interval, with the number and pacing set according to how many sums each individual participant could perform at 80% correct within the time allowed. For the dual-task condition, the letter sequence was presented as a memory preload, then arithmetic verification was performed during a 10-s retention interval followed by recall of the letter sequence. Single- and dual-task conditions were performed in silence or with articulatory suppression. Providing a complete rationale for each set of predictions is outside the scope of the current article, but in summary the TBRS team predicted that there would be a large main effect of dual task and of articulatory suppression on both memory and processing. The Embedded Processing team predicted a large main effect of dual task and an interaction such that articulatory suppression would affect single-task but not dual-task performance. The Multiple Components team predicted that there would be a small dual-task main effect on memory but not on processing, and a large effect of articulatory suppression on memory with the effect being larger for dual task than single task, and no effect on processing. What is clear from the figure is that, although the results for processing were closest to the predictions for multiple components, none of the three sets of predictions was a complete match for the observed results. There were clear main effects of dual task and articulatory suppression on memory but no interaction, and there were no effects of either dual task or articulatory suppression on processing.

This general finding that the observed data matched different aspects of the three contrasting predictions, but not all of the predictions for any one theory, was the case for all 15 experiments that were carried out during the project. All three teams could generate post hoc explanations about how the data could be interpreted to fit with their respective theoretical assumptions for the experiment shown in Figure 10, but none of the experiments provided a complete match with any of the three predicted alternatives.

**Figure 11.**
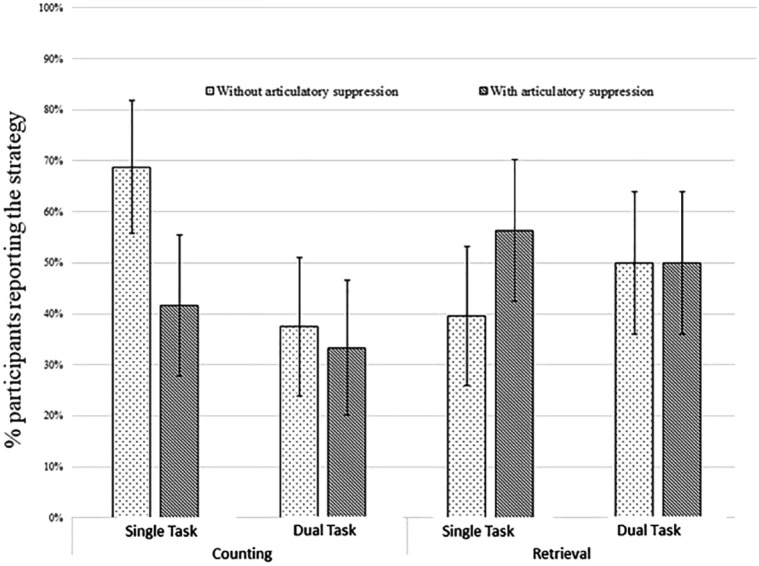
Counting or retrieval strategy reported for arithmetic verification as single task or with letter memory, without/with articulatory suppression. *Source.* Reproduced from Belletier et al. (2023).

As a follow-up to the earlier discussion on the importance of considering variation in cognitive strategies, for the Doherty et al. (2019) experiment shown in Figure 10, at the end of the experimental session, we asked participants how they performed each task in the different experimental conditions. The strategy responses were reported in Belletier et al. (2023), and some of those results for the arithmetic verification task are shown in Figure 11. A new experiment was also reported in that more recent paper in which participants were asked after each trial how they had performed in the task. The results from participant reports were very similar across experiments. The most common strategies to be reported involved either counting (e.g., for the sum 7 + 4 = 12, participants would count 7 – 8 – 9 – 10 − 11—respond false), or retrieval (participants retrieved the correct answer directly from their knowledge of arithmetic). What we found was that, between single- and dual-task conditions, and between without and with articulatory suppression, the number of participants reporting the counting strategy dropped, and reports of the retrieval strategy increased. In other words, many of the participants were changing the way in which they were performing the arithmetic verification task between experimental conditions. This went some considerable way to explaining why for the processing task in Figure 10, there was no impact of dual task, and no impact of articulatory suppression. There were also reports of changes in strategies for the memory task between conditions, although as is clear from Figure 10, memory performance was affected by dual task and articulatory suppression. However, these effects did not interact as had been predicted by all three theoretical perspectives, and appeared to be quite independent. In summary, articulatory suppression resulted in a dramatic reduction in the reports of using articulatory rehearsal for remembering the letter sequences, as might be expected. However, dual task had no impact on the number of participants reporting the use of rehearsal. In contrast, reports of the use of mnemonic strategies increased with articulatory suppression, but decreased under dual-task conditions. A small number of participants also reported using temporal clustering, and visual and acoustic strategies. Some participants reported that they attempted to remember only some of the presented items for recall, so it reduced their memory load, particularly with dual task and with articulatory suppression. These findings support my earlier arguments that multiple cognitive strategies are used by participants for the same task, and demonstrate further that strategies might change for the same task under different experimental conditions. They further emphasise the importance of studying and understanding the cognitive functions that people have available and bring to bear for task performance rather than developing theories of tasks or of patterns of effects that are found in aggregate data.

The extended period of collaboration ensured that each researcher gained a much deeper understanding of the alternative theoretical motivations and goals. None of the teams completely abandoned their theoretical perspectives, but all modified their assumptions and the theories became more similar (Cowan et al., 2020). For example, Cowan et al. (2021) has acknowledged that there may be limited storage of a small amount of domain-specific information separately from the focus of attention. Barrouillet and Camos (2021) have incorporated multiple components in their TBRS framework, which now includes, for example, a phonological buffer, a visuo-spatial buffer, and a motor buffer in addition to motor programmes for articulation. For example, Barrouillet et al. (2021) have shown that for supra-span verbal lists (e.g., lists of seven or more items), there are two strategies involved: one for verbally rehearsing part of the list, and the other for thinking about the remaining items in the list. These two strategies combine to generate overall performance on the task. As a result of acquiring a much greater understanding of the detailed assumptions of these two alternative theoretical perspectives, Logie, Belletier, and Doherty (2021) proposed that one major reason for ongoing debates was that each group was focused on a different level of explanation for their theoretical goals. That is, Embedded Processes tends to focus on the level of explanation that considers the overall capacity of working memory rather than how that overall capacity is achieved, whereas Multiple Components tends to focus on the different level of explanation as to how capacity is achieved rather than overall capacity. TBRS originally focused on overall capacity, but its more recent version (Barrouillet & Camos, 2021) incorporates a range of different contributions to that capacity. The proposal in Logie, Belletier, and Doherty (2021) attempted to resolve the debate by integrating concepts from the three perspectives, and this is summarised in the next, and final section.

## Resolving debate through theory integration

Following completion of the WoMAAC project, Logie, Belletier, and Doherty (2021) suggested that one key difference between the theoretical perspectives might be that different laboratories are using the category label of working memory to refer to different underlying concepts, even though we agreed on our introspections about the everyday activities that working memory supports (Logie et al., 2021a). As noted earlier, Cowan’s primary interest was and is in understanding conscious experience, and this most likely reflects an individual’s personal mental experience of the whole cognitive system functioning in a seamless manner to support cognitive performance on current tasks. As such, the perspective of embedded processes does an excellent job of characterising conscious experience, which is the level of explanation for the theoretical goal. However, as argued earlier, not necessarily all that is active in the brain is necessarily available to consciousness, and our conscious experience might not offer a wholly accurate impression of the underlying organisation of cognition. Moreover, the concept of working memory is a psychological construct, along with other psychological constructs such as attention and executive functions. The verbal labels for these constructs map on to our personal mental experiences, so we assume they have some reality, and this then can drive the design of experiments and interpretation of empirical data. But if our goal is at a different level of explanation rather than to understand conscious experience, we might adopt a different theoretical perspective.

As argued recently by Brick et al. (2022), the matches between the concept of working memory or attention and our intuitions about cognition could be illusory categories that offer a convenient way to group a set of research topics that reflect our conscious experience. In this sense, there is a risk that, despite the large body of empirical data collected over the past century, there is a continuing powerful influence of *introspective psychologizing* (James, 1902) discussed in the first section of this article. Moreover, there is no guarantee that psychological constructs such as working memory or long-term memory will map directly onto specific areas or networks in the brain. They could reflect different modes of operation for a given network or for overlapping brain networks, with each mode serving different functions. For example, some time ago Duncan (2004; Duncan et al., 1997) suggested that the psychological construct of attention reflects biases in activation that shift across a range of brain areas, not the operation of an identifiable brain structure or network. Therefore, the argument that working memory comprises activated long-term memory is not incompatible with suggesting that working memory and long-term memory are conceptually different and have different functional roles in cognition, or that they can be selectively damaged. Because working memory and long-term memory continuously interact in the healthy brain, it is inevitable that there will be overlapping brain networks involved in temporary storage and moment-to-moment cognition, and those involved in long-term storage. Within the range of different levels of cognitive explanations, it does not matter whether what we define to be working memory comprises activated information from what we define to be long-term memory, or that tasks assumed to measure working memory or long-term memory involve different, but interacting brain networks. Debates among cognitive theories might simply reflect different ways to think about psychological constructs that have been shown to be useful to account for a substantial body of behavioural data from healthy and brain damaged individuals, as described in the earlier sections of this article. This does not necessarily mean that they are located in completely different brain areas, or indeed that these psychological constructs will neatly map onto specific brain networks. For example, Sreenivasan and D’Esposito (2019), and Lorenc and Sreenivasan (2021; Lorenc et al., 2018) demonstrated that a broad range of areas in the brain are involved in temporary maintenance for working memory tasks, and not only medial frontal and medial temporal lobe areas (e.g., Kaminski et al., 2017). If the theoretical goal is to understand cognitive function with reference to conscious experience, or to understand cognitive function without reference to consciousness, or to understand brain function and organisation as brain structures and connections, or to understand synaptic growth, or to understand molecular biology, those are different levels of explanation, not competing theoretical frameworks, and it might be very misleading to assume that theoretical constructs developed for one level of explanation are relevant or helpful to support one or other side of a debate at a different level of explanation.

In Logie, Belletier, and Doherty (2021), it was suggested that studies that consider measures of overall working memory capacity are assessing a range of cognitive functions that are working in concert and seamlessly to support performance. The demonstration by Barrouillet et al. (2021) of two contributions to the measure of verbal memory span is a good example of this. To take the analogy of physical performance, we can measure the speed with which an athlete can run 100 m, and at this level of explanation, it gives an overall measure of the health and fitness of the individual. However, it reveals nothing at a different level of explanation, of the various components of the individual’s physiology such as the heart, lungs, and muscles, all of which are required to function and interact with many other bodily functions, including the brain, for successful performance. In an analogous fashion, the brain works as a whole to generate a performance score on a cognitive test, not only those areas of the brain that are seen as more active in one experimental condition compared with another. Moreover, no one task can be process pure, so there are multiple contributions to performance. Any task thought to measure working memory necessarily requires contributions from acquired knowledge and skills to understand the stimulus material, and to understand and follow instructions. So what is assumed to comprise the psychological construct of long-term memory will inevitably be involved in the performance of any working memory task. This is an inherent property of the conceptual outline illustrated for Multiple Components in Figure 1 and of both the Embedded Processes view of activated long-term memory (Cowan et al., 2021) and the TBRS Framework (Barrouillet & Camos, 2021). However, because the multiple contributions to overall performance on a task interact in the healthy brain to support performance, their individual contribution may not be evident in the observed data, but may be revealed when their function is compromised by brain damage or by an experimental manipulation.

**Figure 12.**
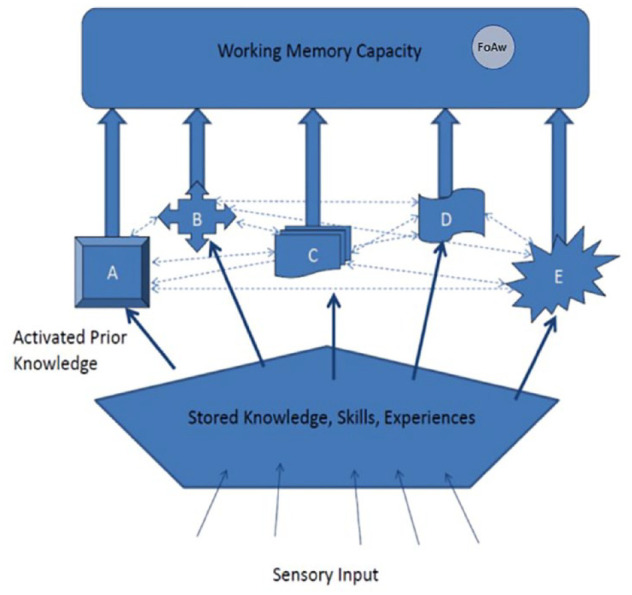
A schematic illustration of a multiple component WM system, developed from Logie (1995, 2003, 2011, 2016, 2018a). Sensory input activates stored knowledge, skills, and experiences. The activated information is made available to a range of domain-specific memory systems and cognitive functions in working memory (A to E in the Figure). Control within WM arises from local interactions (dotted arrows) between domain-specific systems that function in concert in different combinations as required to support task performance. Measures of overall WM capacity assess the cumulative contributions of the interacting domain-specific systems. FoAw = Focus of Awareness, refers to conscious experience of current cognitive functions, not the locus of control. *Source.* Reproduced from Logie, Belletier, and Doherty (2021).

Figure 12 outlines a conceptual framework for the different levels of explanation that might characterise different theoretical perspectives for working memory. The upper section labelled working memory capacity illustrates the level of explanation that is associated with individual differences in measures of working memory span and links with general fluid intelligence (e.g., Engle, 2018; Kane & Engle, 2002; Mashburn et al., 2021) or that assumes working memory comprises activated long-term memory coupled with a focus of attention (e.g., Cowan, 1999, 2005/2016; Cowan et al., 2021). This is the level of explanation that considers overall working memory capacity. The lower part of Figure 12 illustrates a different level of explanation that considers how multiple aspects of cognition interact seamlessly in the healthy brain to provide the overall capacity, just as different aspects of our physiology (e.g., heart, lungs, liver, muscles) operate together to allow for coordinated physical and mental activity in the everyday life of an individual.

In the lower section of the figure, multiple cognitive functions are given the arbitrary labels A to E, each of which has a distinctive role in cognition. But these do not work in isolation. They interact (the dotted lines) in different combinations to support task performance as measured by the overall working memory capacity. For example, components labelled A and B in the figure could represent the phonological store and articulatory rehearsal and these interact to support one strategy for achieving temporary storage of phonological codes. Components D and E could reflect the inner scribe and visual cache, which together can support temporary retention of a small amount of visual and spatial information. Different strategies involve different combinations of components. For example, earlier in this article, I described evidence for the possible use of visual codes to support serial ordered verbal recall. If participants rely solely on subvocal rehearsal, the components A and B in the figure would be used. If they also use visual codes, then components D and E would also be involved in supporting task performance. The interaction between components A, B, D, and E together with relevant material activated from long-term memory could represent the storage of temporary combinations of multiple types of information for which Baddeley (2000; Baddeley et al., 2021) proposed the psychological construct labelled as the Episodic Buffer. The interaction of A, B, C, D, E, and interactions with other cognitive functions such as episodic and semantic memory, language skills, perception, inhibition, task switching, and updating, together would result in overall working memory capacity. In other words, there is a “toolbox” of cognitive functions that people have available, and they use them in different combinations to support performance on the current task.

The most recent version of the TBRS framework (Barrouillet & Camos, 2021) incorporates the combined operation of different cognitive functions, together with the emerging properties of their interaction. As such, TBRS is entirely compatible with the outline framework in Figure 12. The multiple component framework has a focus primarily on the functioning of the individual components and their multiple interactions while placing less emphasis on the overall capacity of the system. Embedded Processes place much greater emphasis on overall capacity. These are different perspectives for the psychological constructs developed for different levels of explanation, not mutually incompatible theories.

What of the homunculus for centralised control? As proposed by Duncan (2004; Erez et al., 2022; Wen et al., 2019; see also Eimer, 2015), the level of activity of different brain structures and networks shifts dynamically between them according to the demands of the task, not the result of a single, centralised control mechanism. As some networks become more active, others become less active and so are less effective for the aspects of task requirements that they support. A key aspect of Figure 12 is that the dotted lines with arrows represent the local interactions between components. What are considered to be the psychological constructs of attentional or executive control are proposed to arise from a very large number of these multiple local interactions, and not from any form of central executive. This feature of the conceptualisation in Figure 12 might be seen as a way of finessing the requirement to explain the psychological constructs of attention, top–down control, or the central executive, but it is entirely compatible with Duncan’s (2004) biased competition model for attentional control. Moreover, there are numerous examples in the natural world of complex, self-organising control arising from multiple local interactions. For example, ant colonies function in this way, with each individual ant having a defined, specialised role, but the different priorities, such as seeking food or defence against a threat, could change the pattern of activity (focus of attention) across the colony, without any form of executive. The coordinated murmurations of starlings, or seemingly coordinated movements of shoals of fish, and of groups of non-human primates, as well as social interactions among humans provide some of the many examples in which local interactions between individuals provide overall control for the group without any form of centralised control (e.g., Eisenreich et al., 2017; Morgan, 1986).

Self-organising principles from multiple local interactions have been used for control of multiple “smart” domestic appliances (Nascimento & Lucena, 2017), and are also being used for decentralised cooperative navigational control of satellites in orbit to stop them from colliding accidentally (Qin et al., 2021). There are now several implementations of computational and cognitive neuroscience models of distributed control (e.g., Barnard, 1999; Hazy et al., 2006, 2007; Lorenc et al., 2018; Lorenc & Sreenivasan, 2021; Vandierendonck, 2016, 2021; Willshaw, 2006). Some time ago, the Hazy et al. (2006) paper was entitled “Banishing the homuculus.” More recently I suggested offering the Central Executive a dignified retirement (Logie, 2016).

## Conclusion

Much of cognitive psychology is fuelled by empirical studies that provide evidence for or against one or the other side of a theoretical debate, and particularly in research on the concept of working memory. This has led to a proliferation of competing theoretical frameworks that aim to account for how humans keep track of, and continually update their mental representations on a moment-to-moment basis to support everyday activities. Rarely, if ever, does this kind of scientific competition result in a winner, so debates self-perpetuate, in some cases for decades, and diversify rather than converge on what is generally agreed to be a scientific advance.

I have argued that some of these debates might have arisen from differences across laboratories in the theoretical goal and the level of explanation being pursued, but also from variation in the strategies that participants may adopt to perform the same tasks. There is a tendency to focus on developing theories of tasks, rather than consider that there are several different ways in which a given task might be performed. So, several alternative theories for a task may be correct, depending on the strategy that participants adopt for task performance. Inter- and intra-subject variation in data patterns tends to be treated as statistical noise rather than as informative for understanding human cognition, and conclusions tend to be based on patterns in aggregate data. These patterns may be informative about how the majority of participants perform a task. However, before drawing final conclusions, the aggregate pattern should be considered in the context of how many participants do not show the group pattern. It is common practice to determine whether individual participants are performing at floor or ceiling on a task, and to omit these data from the analyses. However, rarely is there any detailed consideration of whether participants, whose performance levels are similar to those of the majority, show data patterns that differ from the group averages, perhaps indicating variation in strategy use for performing the task in a different way from the majority. Even a ceiling effect might indicate the use of an alternative strategy that is more effective than that used by other participants, as shown in studies of the effects of expertise on memory (e.g. Ericsson et al. (2018). Multiple examples are described from studies of healthy adults and neuropsychological case studies, demonstrating that conclusions from aggregate data alone may be misleading, and that at least some debates might be resolved by considering the range of cognitive functions that participants have available to perform a task.

It is proposed that adversarial collaboration, in which researchers from different sides of a theoretical debate agree to work within an extended, common project, has the potential to resolve debates through greater mutual understanding of theoretical perspectives, through use of a common methodology, and generation of a common dataset that is trusted by all. An example of an extended adversarial collaboration is described that resulted in three different theoretical frameworks for working memory becoming more similar, and demonstrated the importance of considering variation in strategy use to help resolve some theoretical differences. Finally, a route to theory integration is proposed based on whether the theoretical goal is to account for overall working memory capacity or is at a different level of explanation regarding how that overall capacity might arise from seamless interactions between multiple aspects of cognition that are deployed in different combinations to support task performance.

## Notes

1. A large number of papers and reviews incorrectly have attributed the visuo-spatial sketchpad concept to Baddeley and Hitch (1974), but this concept was developed in other studies (e.g., Baddeley, Grant, et al., 1975) and explored in detail in Baddeley (1986). The label visuo-spatial sketchpad was proposed by Gerry Quinn (Baddeley, 1986, p. 109). See commentary in Logie (2023).

2. The plots of response time against degree of rotation in Figure 4 and for the healthy controls in Figure 5 look different, particularly for rotation angles greater than 100°. Possible accounts for this are beyond the scope of this article. However, it should be noted that the participants in Logie et al. (2011, Figure 4) were young adult university students in the age range of 20–35 years, whereas the healthy participants in Zeman et al. (2010, Figure 5) were chosen to match the case study MX on professional skills and age, and so were all professional architects or surveyors in the age range of 55–76 years.
